# Editor’s Book Table

**Published:** 1870-03

**Authors:** 


### Editor's Book Table.

[NOTE.—All works reviewed in the columns of the CHICAGO MEDICAL JOURNAL may be found in the extensive stock of W. B. Keen and Cooke, whose catalogue of medical books will be sent to any address upon request.]

*Clinical Lectures on the Principles and Practice of Medicine.* By JOHN HUGHES BENNETT, M.D., F.R.S.E., Professor of the Institutes of Medicine, and Senior Professor of Clinical Medicine in the University of Edinburgh, etc., etc. Fifth American (from the Fourth London) edition. With 537 Illustrations on wood. New York: William Wood & Co., 61 Walker Street. 1870. Pp. 1,022.

THE rapid exhaustion of successive reprints of this invaluable work is sufficient evidence of the high favor in which it is held by the profession. Without indorsing all the peculiar conclusions at which Prof. Bennett arrives, we can sincerely say that his earnest advocacy and powerful support of physiological and pathological investigations as the real foundation of rational therapeutics, entitle his writings to the careful study of all physicians desirous of elevating practical medicine above mere empiricism; and even though the teachings of a semi-blind "experience" are still to be obstinately adhered to, Prof. Bennett still shows excellently well how to employ the most approved methods in making up the diagnosis. Suffice it to say, we consider this one of the indispensable books for the physician's library and study.

*Bellevue and Charity Hospital Reports.* 1870. [L. S.] New York: D. Appleton & Co., 90, 92 and 94 Grand Street. 1870. Pp. 416.

WE opened this book expecting to find little more than a mass of tolerably dry historical details and statistics, but were most agreeably disappointed by finding instead a series of fourteen really valuable essays upon various important points: Amputation of the Cervix Uteri — J. E. Taylor, M.D.; Sundry Physical Signs, three papers, by Austin Flint, M.D.; Excision of the Os Calcis, by F. A. Burrall, Jr., M.D.; Injuries of the Ankle Joint, and Mode of Dressing Fractured Clavicle, by Lewis A. Sayre, M.D.; Seventy-three Cases of Hernia, etc., by Frank H. Hamilton, M.D.; One hundred and two Cases of Bright's Disease, by Austin Flint, M.D.; On Some of the Effects of Excessive Intellectual Exertion, by Wm. A. Hammond, M.D., and several others of like interest. It is, as a whole, a valuable contribution to medical literature.

Why do not our Chicago hospitals take the hint, and go and do likewise?

*Personal Beauty; How to Cultivate and Preserve it in accordance with the Laws of Health.* By D. G. Brinton, M.D., Editor "Medical and Surgical Reporter," Philadelphia, etc., etc., and George H. Napheys, M.D., Chief of Medical Clinic of the Jefferson Medical College, etc., etc. Springfield, Mass.: W. J. Holland. 1870. Pp. 346.

Although this book was evidently written for popular use, yet practitioners will find in it many valuable hints not readily to be found in the text-books, or even the more elaborate library volumes. It is written in an easy, flowing style, relieved by frequent anecdote and witticism — just the thing to read after a hard day's work, with the feet upon the fender, and the lips closed on — if you can get it — a first-class *Havana*.

---

### Pamphlets.

*Contributions to Practical Laryngoscopy.* Four Cases of Morbid Growths within the Larynx. Reported by A. Ruppaner, M.D. Plates.

*Spinal Diseases.* Pathology and Treatment of the Backward and Lateral Curvatures. By Julien S. Sherman, M.D., Chicago.

*A Prize Essay on the Medical Activity of the Hemp Plant of North America.* By Dr. Horatio C. Wood, Jr., Professor of Botany, University of Pennsylvania.

*Report of Committee on Consanguineous Marriages.* By Dr. Robert Newman, of New York. Presented to the New York State Medical Society. 1869.

*Dr. Watters' Doctrines of Life.* Reply to London *Lancet*, and Closing Remarks. November, 1869.

*Prize Essay—Acupressure.* The Cash Prize awarded by the New York State Medical Society. 1869. By Joseph C. Hutchinson, M.D., Brooklyn, N. Y.

*The Physical and Medical Topography, including Vital, Manufacturing, and other Statistics of the City of Wheeling, Va.* By James E. Reeves, M.D., City Health Officer, etc., etc.

*Fifth Annual Report of the Directors and Superintendents of the Illinois Institution for the Education of Feeble-minded Children, located at Jacksonville.*

*The Half-Yearly Abstract of the Medical Sciences.* Being a Digest of British and Continental Medicine, and of the Progress of Medicine and the Medical Sciences. Edited by Wm. Domett Stone, M.D., F.R.C.S. (Exam.) Vol. I. January, 1870. Philadelphia: Henry C. Lea. 1870. Pp. 296. $2.50 a year. Single copies, $1.00.

*Medical Department of the Library of the Long Island Historical Society.* An Account of its Formation, with a Catalogue of its Books. Brooklyn. 1870. From C. L. Mitchell, M.D., Brooklyn.

*Transactions of the American Ophthalmological Society.* Sixth Annual Meeting. 1869. Also, of the *American Otological Society.* Second Annual Meeting. 1869.

[Several of the above monographs, etc., will be further noticed as soon as space will permit.]

*The American Journal of Syphilography and Dermatology.* Edited by M. H. Henry, M.D., Surgeon to the New York Dispensary, etc. January, 1870. Published quarterly. New York: F. W. Christem, No. 77 University Place; Lindsay & Blakiston, Philadelphia. $3 a year. Single copies, $1.

THE initial number of this journal is handsomely gotten up, and its contents are as varied and interesting as the specialties to which it is devoted will permit. We wish the enterprising editor and publishers abundant success.

